# Echinacea Supplementation Does Not Impact Aerobic Capacity and Erythropoiesis in Athletes: A Meta-Analysis

**DOI:** 10.3390/nu16131991

**Published:** 2024-06-22

**Authors:** Stephanie Deccy, Callie Bartkowiak, Nathan Rodricks, Kristopher Paultre

**Affiliations:** 1 Department of Family Medicine, University of Miami, Miami, FL 33136, USA; 2 Jackson Health Systems, Miami, FL 33136, USA; 3 Department of Orthopedics and Student Healthcare Clinic, University of Miami Health Systems, Miami, FL 33146, USA; 4 Club Sports, Department of Wellness and Recreation, University of Miami, Miami, FL 33146, USA

**Keywords:** dietary supplementation, performance enhancement, athletic performance

## Abstract

Athletes are increasingly relying on natural supplements to improve athletic performance. Echinacea, a common herbal supplement, has been studied for its potential erythropoietin-enhancing effects, with mixed results in the literature. The purpose of this meta-analysis is to determine whether echinacea supplementation has erythropoietic or ergogenic effects in athletes. A search strategy was developed to identify trials studying the impact of echinacea supplementation on erythropoiesis and maximal oxygen uptake. The database search yielded 502 studies, 496 of which were excluded in the two-reviewer screening process. Six studies with a total of 107 athletes were included in the analysis. For hemoglobin and hematocrit levels, there were small, positive effect sizes when comparing the difference in pre- and post-intervention levels between the echinacea and placebo groups, at 0.38 (*p* = 0.02, 95% CI −0.04–0.80, *I*^2^ = 70%) and 0.34 (*p* < 0.01, 95% CI −0.10–0.78, *I*^2^ = 86%), respectively, though they did not reach statistical significance. There was also no statistically significant change in erythropoietin (effect size −0.29, *p* = 0.05, 95% CI −0.75–0.17, *I*^2^ = 67%) or maximal oxygen uptake (effect size −0.20, *p* = 0.95, 95% CI −0.60–0.21, *I*^2^ = 0%). Echinacea supplementation did not influence erythropoietin, hemoglobin, hematocrit, or maximal oxygen uptake in athletes; however, the evidence base is limited.

## 1. Introduction

*Echinacea purpurea* is a member of the sunflower family that has long been used for its anti-inflammatory and pro-immunity effects, with a myriad of uses in holistic medicine ranging from a common cold remedy to anxiolytic psychotropic effects [1]. Echinacea’s most well-studied application is for the prevention and treatment of the common cold; a systematic review encompassing 14 studies and 1600 patients concluded that echinacea proved beneficial in decreasing both the incidence and duration of the common cold [2]. While echinacea’s role in immune health is well-characterized, it has also been investigated for possible ergogenic and erythropoietic properties. The mechanism of echinacea’s impact on erythropoiesis is poorly understood, however the basic science literature has shown an increase in pro-inflammatory cytokines (i.e., IL-1, TNF-alpha, IL-6) and oxygen radicals by macrophages exposed to echinacea vs. control [3,4]. Erythropoiesis is influenced by cytokines such as IL-6 and TNF-alpha, which possess pro-inflammatory qualities; the body’s inflammatory response has been studied as a catalyst for an alternative “stress erythropoiesis” pathway for increased red blood cell production during periods of physical stress [5,6]. Animal studies have demonstrated convincing findings with respect to echinacea’s erythropoietic potential; for example, in a double-blind, placebo-controlled crossover study conducted in horses, 42 days of echinacea supplementation to the horse feed increased both the size and concentration of peripheral red blood cells as well as hemoglobin (Hb) concentration compared to the placebo group [7]. In another study in rabbits, a dose-dependent, statistically significant increase in Hb concentration was observed during 3 months of echinacea supplementation; compared to the control rabbit population Hb level of 10.56 mg/dL, the low, medium, and high-dose echinacea groups were found to have average Hb levels of 11.19, 11.72, and 13.11 mg/dL, respectively [8].

Erythropoietin (EPO) and erythropoiesis-stimulating agents (ESAs) have been shown to positively influence aerobic capacity via the stimulation of red blood cell production, resulting in improved oxygen delivery to peripheral tissue. Maximal oxygen uptake (VO_2_ Max), which is the maximal rate at which oxygen is used by muscle tissue during exercise, is a commonly used metric to quantify aerobic fitness and has predictive value with respect to race performance in runners [9,10,11]. EPO and its downstream effects on Hb and hematocrit (Hct) levels are directly linked to VO_2_ Max, particularly for trained endurance athletes, as VO_2_ Max in muscle tissue has been shown to change from utilization limitation to diffusion limitation in response to endurance training [12]. A recent meta-analysis including 10 studies with 238 patients demonstrated a benefit of EPO supplementation compared to placebo across a variety of athletic performance metrics including clinical measures such as hematological changes and pulmonary capacity as well as performance-related metrics like maximal power output and time to exhaustion; these effects were observed predominantly during maximal exercise intensities [13]. Consequently, EPO and ESAs have garnered particular attention in the athletic community as a means of performance enhancement, with the World Athletic Anti-Doping Agency (WADA) designating substances known to increase EPO as banned in and out of competition under substance category S2, class 1 [14]. Further complicating WADA’s efforts for clean sport, novel, effective, and increasingly difficult to detect ESAs are being developed [15].

As a result, athletes are increasingly relying on supplements for a safe, natural, and WADA-compliant alternative to procure a competitive advantage, with 45% of a sample of NCAA Division I athletes reporting regular supplement use; this number increases in endurance athletes, with 78% of a sample of elite college endurance runners reporting supplement use during training and competition [16,17]. In a study of Canadian high-performance athletes, a staggering 87% endorsed using three or more supplements in the past 6 months [18]. Echinacea supplementation provides an attractive option for performance enhancement as a well-tolerated natural supplement. However, the literature regarding echinacea’s erythropoietic effects in human clinical trials offers mixed results. For example, Whitehead et al. (2012) found significant increases in EPO, VO_2_ Max, and running economy after 4 weeks of echinacea supplementation in a small sample of healthy young men; conversely, several other controlled trials have reported no change in these outcomes [19,20,21,22]. In addition, a recent literature review including five randomized controlled trials concluded that echinacea does not have EPO- or performance-enhancing qualities [23]. Importantly, further research has been conducted since this review was published in 2016, and a meta-analysis of the literature has not been conducted to date. The purpose of the present study is to understand whether echinacea supplementation enhances aerobic capacity and erythropoiesis through its effect on EPO, Hb, and Hct levels in the blood, as well as its impact on athlete VO_2_ Max.

## 2. Materials and Methods

This meta-analysis was registered with Prospero prior to initiation of research PROSPERO 2023 CRD42023437889 Available from: https://www.crd.york.ac.uk/prospero/display_record.php?ID=CRD42023437889. Accessed on 19 June 2024.

### 2.1. Search Strategy

**Figure 1:**
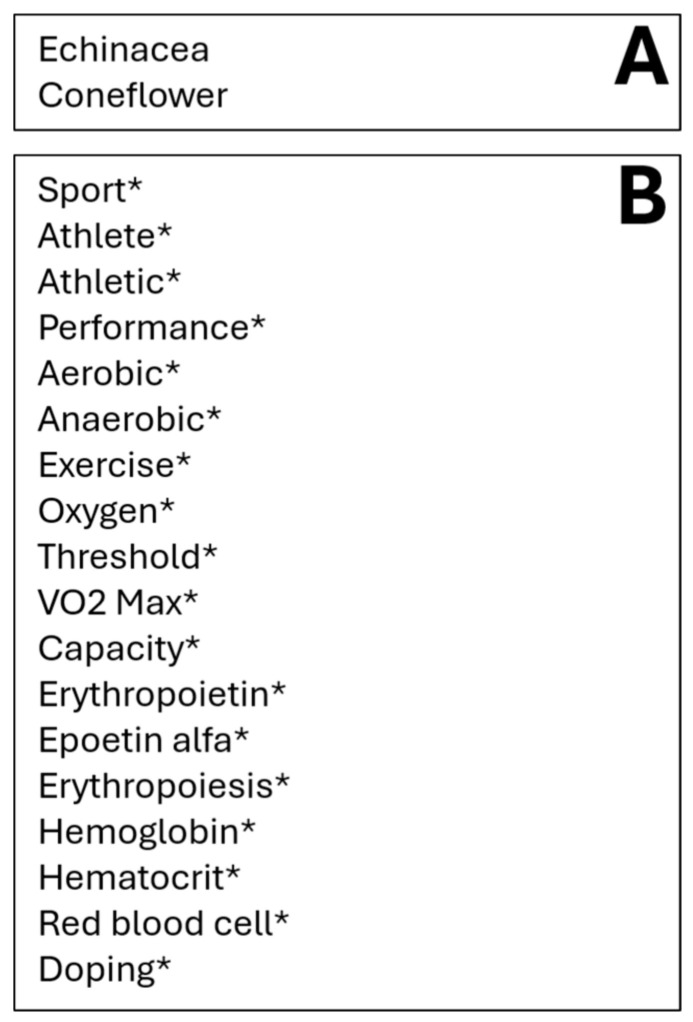
The search strategy employed was the following: (terms in Box (**A**) combined using OR present in title/abstract) AND (terms in Box (**B**) combined using OR present in all fields). * Indicates that the term includes multiple spellings or endings of a word.

To capture all articles relevant to echinacea supplementation and sports performance, a search strategy was developed based on the General Methods for Cochrane reviews. In accordance with the Cochrane guidelines, we included the use of synonyms, related terms, and variant spellings in our terms (i.e., hemoglobin and haemoglobin) and employed the use of Boolean operators. Search strategies documented by previous literature reviews that identified publications related to echinacea were built upon [2]. The search terms “Echinacea” OR “coneflower” in the Title/Abstract field were combined using AND Boolean operator with the following terms in all fields: sport, athlete, athletic, performance, aerobic, anaerobic, exercise, oxygen, threshold, VO_2_ Max, capacity, erythropoietin, epoetin alfa, erythropoiesis, hemoglobin, hematocrit, red blood cell, and doping (Figure 1). 

Four databases (PubMed, CINAHL, Embase, and SPORTDiscus) were queried. Filters for “Human Subjects” and “Clinical Trial” study type were used as available in each database due to a high volume of animal studies and irrelevant study types (i.e., reviews, opinion articles, book chapters) identified during search strategy development. Each database query included all studies up to the date of the search (15 July 2023).

### 2.2. Study Screening and Data Extraction

Screening for relevant articles was conducted based on title and abstract using the systematic review management system Covidence (Covidence systematic review software, Veritas Health Innovation, Melbourne, Australia. Available at www.covidence.org). All records were screened by two reviewers (S.D., C.B.). Disputes were reviewed by a third author (K.P.). Following the initial screen for relevance, each remaining record was further assessed to determine whether the study met the inclusion criteria. Criteria for inclusion in the analysis included all randomized, placebo-controlled or controlled pre-post trials that evaluated the impact of echinacea supplementation on EPO, Hb, Hct, or VO_2_ Max in a study population of adult (age > 18) humans. The included studies underwent data extraction for variables of interest related to the study design (dosage of echinacea supplementation, length of supplementation, study population and demographics) as well as outcome measures of interest (pre- and post-intervention EPO, Hb, Hct, and VO_2_ Max). In addition, a CONSORT score was calculated for all studies based on the 25-item 2010 CONSORT Checklist, which is a guideline for reporting clinical trial data in a transparent and reproducible manner [24,25]. Each checklist item was assigned equal weight (1 point). Risk of bias was also evaluated for each study using the Cochrane Risk of Bias Tool [26].

### 2.3. Statistical Analysis

The mean difference for each outcome of interest was calculated based on the reported pre- and post-intervention means. The standard deviations for each pre- and post-intervention outcome were pooled. Next, the difference in change in each outcome of interest was compared between study and control populations. The magnitude of difference between control and treatment groups was measured using Hedges method for effect size (Hedges’ g) due to the small sample size within studies. Studies that did not include the outcome of interest for a particular analysis were excluded from that analysis. A fixed effects model was used to analyze the data given the small number of studies included in the analysis (*n* = 6) [27]. With respect to the VO_2_ Max outcome, one study (Bellar et al., 2014 [22]) did not include a control group. For this study, the control groups of the other studies were averaged as a surrogate for the control population and given equal weight to the intervention group. In addition, one study (Whitehead et al., 2012 [19]) reported the VO_2_ Max outcome as a percentage change from baseline; consequently, the raw data for the post-intervention VO_2_ Max were extrapolated from the reported baseline. Two studies (Whitehead et al., 2007 [28], Whitehead et al., 2012 [19]) included the same patient population; consequently, the patients were only included in analysis once for each outcome of interest. Stevenson et al. [29] studied double-dose echinacea supplementation (16,000 mg) vs. 8000 mg vs. placebo; for simplification of the analyses and for improved comparison between studies, the 16,000 mg group was excluded from the present study. Analyses were conducted using Meta-Mar (v3.5.1), a free online meta-analysis service, and repeated using R Statistical Software (v4.3.3; R Core Team 2024) using the “meta” analysis package [30].

## 3. Results

**Figure 2:**
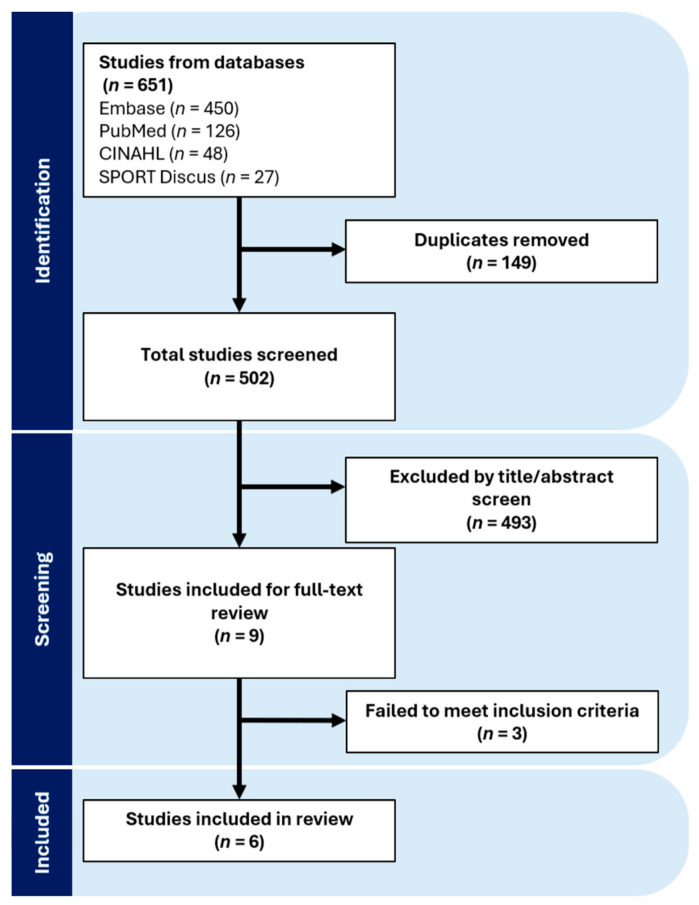
PRISMA Flow Diagram. Adapted from Covidence (Covidence systematic review software, Veritas Health Innovation, Melbourne, Australia. Available at www.covidence.org).

The search strategy yielded 502 studies for screening once duplicates (*n* = 154) were removed. Of these, 493 were excluded based on title and abstract screening. Nine studies underwent full text review with three studies failing to meet inclusion criteria. After screening and full text review, six studies were included in the final analysis (Figure 2).

Combining the participants within all included studies, a total of 107 athletes were considered in the analysis. The length of supplementation between pre- and post-intervention measurements varied between study, ranging from 28 to 42 days. All studies dosed the echinacea at 8000 mg daily. The included studies are summarized in Table 1.

**Figure 3:**
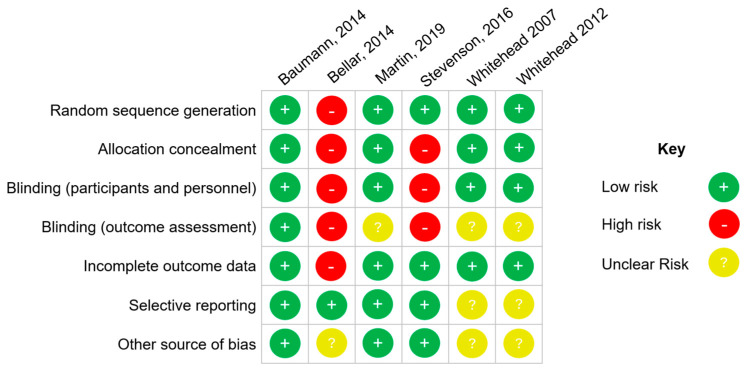
Cochrane risk of bias [19,20,21,22,23].

The average CONSORT grade for all studies was 13. The Cochrane risk of bias for each study are summarized in Figure 3.

Outcome data by study are summarized in Table 2.

**Figure 4:**
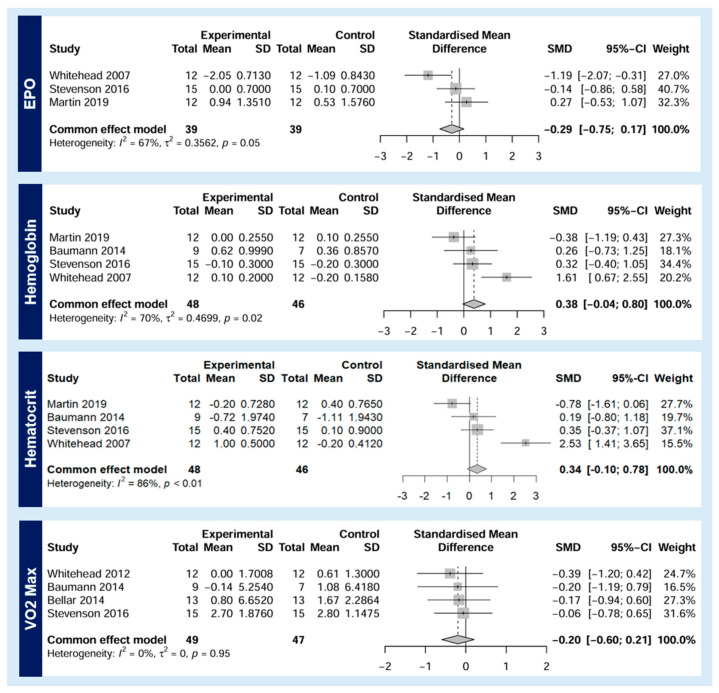
Forest plots by outcome of interest [19,20,21,22,28,29].

With respect to EPO, the small, negative effect size of −0.29 (*p* = 0.05, 95% CI −0.75–0.17, *I*^2^ = 67%) demonstrated no difference between the echinacea and placebo groups. There were small, positive effect sizes when comparing the difference in pre- and post-intervention Hb and Hct levels between the echinacea and placebo groups, at 0.38 (*p* = 0.02, 95% CI −0.04–0.80, *I*^2^ = 70%) and 0.34 (*p* < 0.01, 95% CI −0.10–0.78, *I*^2^ = 86%), respectively; however, this did not reach statistical significance. Change in VO_2_ Max was not significantly different between echinacea supplementation and control groups across any of the included studies (effect size −0.20, *p* = 0.95, 95% CI −0.60–0.21, *I*^2^ = 0%). Forest plots for each outcome of interest are included in Figure 4.

## 4. Discussion

The purpose of the present study was to determine whether echinacea supplementation increases EPO, Hb, Hct, or VO2 Max in athletes based on a meta-analysis of the contemporary literature. The findings indicate that echinacea supplementation does not increase these parameters after 4–6 weeks of daily, high-dose supplementation in a sample of both recreational and endurance-trained athletes. While increases in Hb and Hct neared statistical significance with the small, positive effect sizes observed and the confidence intervals of the SMD including 0 by small margins of −0.04 and −0.10, respectively, the clinical significance of a nominal increase to Hb or Hct of the order of 0.1 g/dL is unconvincing and driven primarily by a single study population. By comparison, altitude training, a well-established performance-enhancer in aerobic sport, has previously demonstrated a 150% increase in EPO level and over 3% increase in Hb concentration, which would be more on the order of 0.5 g/dL [31,32]. Notably, Whitehead et al. (2007) reported a statistically significant (*p* < 0.001) increase in EPO from baseline in the echinacea group observed on days 7, 14, and 21 of the study by 44%, 63%, and 36%, respectively; this change from baseline was not reported after the full 28-day study period which was the only timepoint considered in the present analysis [28]. One other study included in the analysis assessed outcomes at several time points across the study period; however, their data demonstrated no differences between placebo and echinacea supplementation groups across the 14- or 35-day data collection points [29]. All other included studies did not collect blood samples or reported results at more granular time points during the intervention period, limiting the ability to assess the impact of echinacea on a shorter time frame. Accordingly, it is unclear whether echinacea supplementation may provide a short-term benefit that is not sustained after 4–6 weeks of supplementation, and that athletes may attain some benefit with short-term echinacea supplementation prior to competition.

Consistent with a broader trend in the sports medicine literature, the data collection methodology for and definition of VO_2_ Max varied widely between studies. The gold standard of VO_2_ Max measurement is the observation of a plateau of VO_2_ despite the increasing work rate [33]. Of the four studies that considered the impact of echinacea on athlete VO_2_ Max, only two incorporated the gold standard in their definition; however, the gold standard was included as one of several possible criteria that, if met by the athlete, would quantify the athlete’s VO_2_ Max. Other criteria included were the VO_2_ observed once the participant achieved age-predicted maximum heart rate or the VO_2_ at a particular respiratory exchange rate cutoff. One study used the Bruce protocol, which estimates VO_2_ Max using time spent on the treadmill during a heart rate-based assessment [34]. Using secondary metrics such as these to define VO_2_ Max has been shown to diminish the accuracy of the test; there is most concern for inaccuracy in subjects who are exercise-naïve and may fail to reach their exertional capacity [35]. In contrast, young, active participants such as the participants in the present study have been shown to successfully achieve their full volitional capacity in self-paced incremental exercise tests, making these secondary metrics more reliable in estimating their true VO_2_ Max [36]. Furthermore, if an athlete achieved VO_2_ Max using one cut-off criterion at pre-supplementation (i.e., VO_2_ plateau) and met a distinct cut-off criterion (i.e., age-graded maximum heart rate) at the post-supplementation study visit, the results could be influenced by these differences.

The magnitude of the athletes’ echinacea supplementation may have also impacted the results. For example, the 8000 mg per day of echinacea supplementation that was used across all the included studies translated into taking 20 capsules daily of 400 mg of echinacea, which is the standard over-the-counter available dose. While dosing varies wildly for echinacea’s well-studied use for common cold prevention, 200–400 mg three times daily is the most commonly used dosing regimen, representing a 600–1200 mg daily dose. By comparison, all participants included in the analysis consumed 6–13 times this dose range, demonstrating the significant scale of the daily supplementation [2]. The homeopathic principle of hormesis, in which some homeopathic remedies have been shown to be beneficial at lower doses and detrimental or even toxic at higher ones, may have contributed to the observed lack of response in these athletes who were taking comparative mega doses of echinacea [37]. Given that no studies in the literature explored any dose under 8000 mg per day of echinacea, it cannot be determined whether lower doses of echinacea may impact the outcomes of interest.

Both recreational and trained or above-average fitness level endurance athletes were included in the meta-analysis; of the six studies, half were conducted in trained athletes, accounting for 70 (65%) of the 107 total participants. EPO has been shown to provide the most benefit at maximal exercise intensities, which may be more easily achieved and studied in elite athletes, whose VO_2_ Max levels generally greatly exceed those of recreational athletes [38]. Continuing the parallel drawn to altitude training, limited evidence suggests that there may be a physiological difference in elite vs. recreational athlete response to altitude [39]. Consequently, trained athletes may have a more pronounced performance response to more subtle changes in physiologic parameters such as EPO, Hb, and Hct levels. Due to the low sample size, the present study did not compare trained- vs. recreational-athlete response to echinacea supplementation; higher powered studies of trained endurance athletes are needed to determine whether echinacea may provide a performance advantage in this sub-population of athletes.

Also notable is the gender disparity in the athletes studied. This phenomenon is not uncommon in sports medicine research; a recent large-scale systematic review of the sports medicine literature reported significant inequalities in the study of female athletes when compared to their male counterparts, with a staggering 70% of the 669 included studies failing to include female athletes [40]. In the present sample, only one study (Stevenson et al.) included female athletes, such that only 15 out of the 107 of the total participants (14%) identified as female. The inclusion of female athletes is imperative to reducing gender disparities in the literature and is particularly important to investigations of supplement use given reported sex differences in athlete supplementation use and the growing number of female athletes throughout all levels of sport [41,42].

The present review has several strengths including a rigorous search strategy implemented across four databases, study screening by multiple co-authors, and cross-checking of data extraction. Limitations to the present study include the relatively small sample size of 107 athletes across the six included studies, with few female athletes included in the sample. The studies generally had a low risk of bias according to the Cochrane risk of bias. However, the average CONSORT checklist score of the included studies was 13, suggesting that, while the overall adherence to the CONSORT guidelines in this area of research is low, it is comparable to other areas of medical research which have reported similar average CONSORT checklist scores [43]. To better characterize the impact of echinacea on erythropoiesis and sports performance, future studies may focus on shorter time frames of supplementation, with a wider range of doses studied. Importantly, future studies would benefit from larger sample sizes to increase statistical power. The evidence base would also benefit from studies with more gender inclusivity.

## 5. Conclusions

Echinacea supplementation does impact erythropoiesis, as measured by EPO, Hb, or Hct levels, and does not improve aerobic performance as measured by VO_2_ Max, though the research base is limited. Given the widespread use of supplements in sport, more resources should be allocated to explore the impact of echinacea and other substances on sports performance metrics.

## Figures and Tables

**Table 1:** Study design of included studies.

Study	*n*	Population	Female *n*	Intervention	Length of Intervention	Intervention *n*	Placebo *n*	Outcomes of Interest
Baumann et al., 2013 [20]	16	distance runners	not reported	8000 mg daily ech	42 days	9	7	VO_2_ Max, Hb, Hct
Stevenson et al., 2016 [29]	30	endurance-trained athletes	15	8000 mg daily ech	35 days	15	15	VO_2_ Max, Hb, Hct, EPO
Martin et al., 2019 [21]	24	above-average aerobic fitness	0	8000 mg daily ech	42 days	12	12	Hb, Hct, EPO
Whitehead et al., 2007 * [28]	24	recreational athletes	0	8000 mg daily ech	28 days	12	12	Hb, Hct, EPO
Whitehead et al., 2012 * [19]	24	recreational athletes	0	8000 mg daily ech	28 days	12	12	VO_2_ Max
Bellar et al., 2014 [22]	13	recreational athletes	0	8000 mg daily ech ^†^	30 days	13	0	VO_2_ Max

* Studies completed using same participants; ^†^ no placebo group.

**Table 2:** Outcome data by study.

	Echinacea Group								Placebo Group							
	EPO mean (SD)		Hb mean (SD)		Hct mean (SD)		VO2 Max mean (SD)		EPO mean (SD)		Hb mean (SD)		Hct mean (SD)		VO2 Max mean (SD)	
	pre	post	pre	post	pre	post	pre	post	pre	post	pre	post	pre	post	pre	post
Baumann 2013 [20]	-	-	14.93 (1.27)	15.55 (0.80)	43.57 (2.38)	42.85 (1.46)	67.37 (4.62)	67.23 (5.82)	-	-	15.47 (0.9)	15.83 (0.7)	44.61 (2.4)	43.5 (1.34)	65.17 (6.60)	66.25 (6.23)
Stevenson 2016 [29]	6.2 (0.6)	6.2 (0.8)	14.4 (0.3)	14.3 (0.3)	42.4 (0.8)	42.8 (0.7)	59.3 (1.95)	62 (1.80)	9.7 (0.8)	9.8 (0.6)	14.7 (0.3)	14.5 (0.3)	43.1 (0.9)	43.2 (0.9)	61.0 (1.45)	63.8 (1.50)
Martin 2019 [21]	7.92 (1.13)	8.86 (1.54)	14.8 (0.3)	14.8 (0.2)	42.9 (0.9)	42.7 (0.5)	-	-	9.21 (1.3)	9.74 (1.81)	15.0 (0.3)	15.1 (0.2)	43.2 (0.9)	43.6 (0.6)	-	-
Whitehead 2007/2012 [19,28]	12.37 (0.87)	10.32 (0.51)	14.5 (0.2)	14.6 (0.2)	41.9 (0.5)	42.9 (0.5)	43.8 (1.70)	43.8 (1.70) *	10.63 (0.68)	9.54 (0.98)	14.7 (0.1)	14.5 (0.2)	42.5 (0.3)	42.3 (0.5)	40.7 (1.3)	41.31 (1.3) *
Bellar 2014 [22]	-	-	-	-	-	-	51 (6.8)	51.8 (6.5)	-	-	-	-	-	-	x	x

* = extrapolated based on % change graph; x = did not include a control population; - = did not study this outcome. SD = standard deviation.

## Data Availability

No new data were created in this study. Table 2 contains the original data from the studies included in the analysis, which are cited appropriately throughout the text. Further inquiries can be directed to the corresponding author.
